# Crystal structure, mutational analysis and RNA-dependent ATPase activity of the yeast DEAD-box pre-mRNA splicing factor Prp28

**DOI:** 10.1093/nar/gku930

**Published:** 2014-10-10

**Authors:** Agata Jacewicz, Beate Schwer, Paul Smith, Stewart Shuman

**Affiliations:** 1 Molecular Biology Program, Sloan-Kettering Institute, New York, NY 10065, USA; 2 Microbiology and Immunology Department, Weill Cornell Medical College, New York, NY 10065, USA

## Abstract

Yeast Prp28 is a DEAD-box pre-mRNA splicing factor implicated in displacing U1 snRNP from the 5′ splice site. Here we report that the 588-aa Prp28 protein consists of a trypsin-sensitive 126-aa N-terminal segment (of which aa 1–89 are dispensable for Prp28 function *in vivo*) fused to a trypsin-resistant C-terminal catalytic domain. Purified recombinant Prp28 and Prp28-(127–588) have an intrinsic RNA-dependent ATPase activity, albeit with a low turnover number. The crystal structure of Prp28-(127–588) comprises two RecA-like domains splayed widely apart. AMPPNP•Mg^2+^ is engaged by the proximal domain, with proper and specific contacts from Phe194 and Gln201 (Q motif) to the adenine nucleobase. The triphosphate moiety of AMPPNP•Mg^2+^ is not poised for catalysis in the open domain conformation. Guided by the Prp28•AMPPNP structure, and that of the *Drosophila* Vasa•AMPPNP•Mg^2+^•RNA complex, we targeted 20 positions in Prp28 for alanine scanning. ATP-site components Asp341 and Glu342 (motif II) and Arg527 and Arg530 (motif VI) and RNA-site constituent Arg476 (motif Va) are essential for Prp28 activity *in vivo*. Synthetic lethality of double-alanine mutations highlighted functionally redundant contacts in the ATP-binding (Phe194-Gln201, Gln201-Asp502) and RNA-binding (Arg264-Arg320) sites. Overexpression of defective ATP-site mutants, but not defective RNA-site mutants, elicited severe dominant-negative growth defects.

## INTRODUCTION

Yeast pre-mRNA splicing (1–3) starts with the assembly of a complex comprising the U1 snRNP bound at the intron 5′ splice site and the Msl5•Mud2 heterodimer engaged at the intron branchpoint. Cross-intron interactions between the U1 snRNP and Msl5•Mud2 establish a scaffold for recruitment of the U2 snRNP to the branchpoint. The U1 snRNP is ejected from the pre-mRNA•U1•U2-containing spliceosome when the U5•U4•U6 tri-snRNP complex joins *en route* to forming a pre-mRNA•U2•U5•U6 spliceosome. Dissociation of U1 snRNP is thought to be triggered by the DEAD-box protein Prp28 (4,5), acting to disrupt the short U1:5′ splice site RNA duplex or remodel protein–RNA contacts at the 5′ splice site (or both).

This hypothesis regarding Prp28 function is predicated on findings that the essentiality of Prp28 for vegetative growth can be bypassed by mutations in the essential U1 snRNP subunits Yhc1, Prp42, Snu71 and SmD3 and by specific U1 snRNA mutations located within and flanking the segment that base-pairs with the intron 5′ splice site (4,6–8). These genetic interactions imply that the need for Prp28 during U1 snRNP ejection from the early spliceosome is alleviated by certain alterations that weaken U1•5′ splice site contacts.

What remains unclear is how Prp28 elicits a conformational switch in the spliceosome. The 588-aa Prp28 polypeptide is a member of the DEAD-box family (9) and includes the 12 conserved amino acid sequence motifs that define this clade of nucleic acid-dependent NTP phosphohydrolases (10,11). Therefore, it has long been presumed that Prp28 possesses NTPase activity. However, there are no data extant that document this activity, notwithstanding that the titles of several publications designate yeast or human Prp28 to be an ATPase (12,13) or a helicase (14). To the contrary, in the initial isolation of Prp28 from yeast extracts, Strauss and Guthrie were ‘unable to obtain convincing results’ of an ATPase activity or RNA helicase activity that co-purified exclusively with the Prp28 polypeptide (15). More recently, Yang *et al.* (12) reported that purified recombinant yeast Prp28 had no detectable ATPase activity and Möhlmann *et al.* (14) detected no ATPase activity with purified recombinant human Prp28.

The current view of DEAD-box protein function (10,11) does not insist that family members act as nucleic acid translocases powered by iterative NTP hydrolysis. Rather, there is a more nuanced appreciation that DEAD-box proteins can act locally to affect RNA conformation and to either weaken or strengthen RNA–protein complexes. In the words of Putnam and Jankowsky (11), ‘it is clear that the molecular basis for the cellular function of each [DEAD-box protein] lies in its biochemical capacity’.

In the present study, we demonstrate that yeast Prp28 is a *bona fide* RNA-dependent ATPase, albeit with a low turnover number. We report the crystal structure of a truncated ATPase-active Prp28-(127–588) protein in a complex with AMPPNP and magnesium that exemplifies a wide-open domain arrangement that is not poised for ATP hydrolysis. Guided by our Prp28•AMPPNP structure, and that of the homologous DEAD-box protein *Drosophila* Vasa in complex with AMPPNP•Mg^2+^ and RNA (16), we survey the effects of alanine substitutions on Prp28 function *in vivo*.

## MATERIALS AND METHODS

### Recombinant Prp28 proteins

The *PRP28* open reading frame (ORF) was amplified from *Saccharomyces cerevisiae* genomic DNA by polymerase chain reaction (PCR) with primers that introduced a BamHI site at the start codon and an XhoI site immediately after the stop codon. A truncated ORF encoding Prp28-(127–588) was generated by PCR amplification with a sense-strand primer that introduced a BamHI site overlying the codon for Asn127. A missense Asp341-to-Ala change was introduced into the Prp28-(127–588) ORF by PCR with mutagenic primers. The PCR products were digested with BamHI and XhoI and inserted between the BamHI and XhoI sites of pET28b-His_10_Smt3 to generate expression plasmids pET-His_10_Smt3•Prp28, pET-His_10_Smt3•Prp28-(127–588) and pET-His_10_Smt3•Prp28-(127–588)-D341A encoding the full-length Prp28, Prp28-(127–588) and Prp28-(127–588)-D341A polypeptides, respectively, fused to an N-terminal His_10_Smt3 tag. The inserts in each plasmid were sequenced to verify the fusion junctions and to ensure that no unwanted coding changes were introduced during amplification and cloning.

The Prp28 expression plasmids were transformed into *Escherichia coli* BL21(DE3) CodonPlus cells. Cultures (2-l) amplified from single transformants were grown at 37°C in Terrific Broth containing 50 μg/ml kanamycin and 0.4% (v/v) glycerol until the *A*_600_ reached 0.6. The cultures were chilled on ice for 1 h, adjusted to 2% (v/v) ethanol and 0.3 mM isopropyl-β-D-thiogalactopyranoside (IPTG) and then incubated for 20 h at 17°C with constant shaking. All subsequent steps of purification were performed at 4°C. Cells were harvested by centrifugation and resuspended in 40 ml of buffer A (50 mM Tris-HCl, pH 8.0, 500 mM KCl, 15 mM imidazole, 10% glycerol) containing one protease inhibitor cocktail tablet (Roche). The cells were lysed by sonication and the insoluble material was removed by centrifugation at 38 000 g for 30 min. Supernatants were mixed for 1 h with 3 ml of His60-Ni Superflow Resin (Clontech) that had been equilibrated with buffer A. The resins were collected by centrifugation, then resuspended in 25 ml of buffer A containing 2 M KCl. The washed resins were centrifuged again, resuspended in 25 ml of buffer A and then poured into gravity-flow columns. The columns were step-eluted with 300 mM imidazole in buffer A. The polypeptide compositions of the eluate fractions were monitored by sodium dodecyl sulphate-polyacrylamide gel electrophoresis (SDS-PAGE) and the peak fractions containing each recombinant protein were pooled. The His_10_Smt3 tags were cleaved by supplementing the protein solutions with Smt3-specific protease Ulp1 (at a Prp28:Ulp1 molar ratio of 1000:1) and dialyzing them overnight against 1000 ml of buffer B (20 mM Tris-HCl, pH 8.0, 5% glycerol) containing 250 mM KCl for full-length Prp28 or 100 mM KCl for Prp28-(127–588). The tag-free Prp28 proteins were separated from His_10_Smt3 by applying the dialysates to 3-ml His60-Ni columns that had been equilibrated with buffer B. The flow-through fractions containing tag-free Prp28 were mixed with an equal volume of buffer B, then applied to pre-packed 5-ml heparin-Sepharose columns (GE Healthcare) equilibrated in buffer C (25 mM Tris-HCl, pH 8.0, 1 mM DTT, 1 mM EDTA, 2.5% glycerol) containing 125 mM KCl for full-length Prp28 or 50 mM KCl for Prp28-(127–588). The bound proteins were eluted with a 75-ml linear gradient of 125 mM to 1000 mM KCl in buffer C for full-length Prp28 and 50 mM to 1000 mM KCl in buffer C for Prp28-(127–588). Peak fractions containing Prp28 were pooled and subjected to gel filtration through a 125-ml Superdex-200 column (GE Healthcare) equilibrated in 10 mM Tris-HCl, pH 8.0, 3 mM DTT, 1 mM EDTA, 3% glycerol and either 250 mM KCl for full-length Prp28 or 150 mM KCl for Prp28-(127–588). Peak fractions were pooled, concentrated by centrifugal ultrafiltration to 8 mg/ml and stored at −80°C. Protein molar concentrations were determined from the *A*_280_ measured with a Nanodrop spectrophotometer (Thermo Scientific), applying an extinction coefficient of 60850 M^−1^ cm^−1^ for full-length Prp28 and 48360 M^−1^ cm^−1^ for Prp28-(127–588), as calculated using Protparam.

### Preparation of SeMet-Prp28-(127–588)

The pET28-His_10_Smt3-Prp28-(127–588) plasmid was transformed into *E. coli* B834(DE3). A single transformant was inoculated into 10 ml of LB medium containing 50 μg/ml kanamycin and incubated for 8 h at 37°C. The bacteria were harvested by centrifugation and then resuspended in 200 ml of complete LeMaster medium containing 50 μg/ml kanamycin and 50 μg/ml selenomethionine (SeMet; L-(+)-enantiomer; ACROS Organics). After overnight incubation at 37°C, the culture volume was increased to 8 l with fresh LeMaster medium (+kanamycin +SeMet) and growth was continued at 37°C with constant shaking until the *A*_600_ reached 0.6. The culture was placed on ice for 1 h, adjusted to 0.3 mM IPTG and 2% ethanol and then incubated for 18 h at 17°C with continuous shaking. Cells were harvested by centrifugation, and the pellet was stored at −80°C. The SeMet-Prp28-(127–588) protein was purified as described above for native Prp28-(127–588). Peak gel filtration column fractions were pooled, concentrated by centrifugal ultrafiltration to 12 mg/ml in 10 mM Tris-HCl (pH 8.0), 5 mM DTT, 150 mM KCl and stored at −80°C.

### Limited proteolysis of Prp28

Reaction mixtures (10 μl) containing 50 mM Tris-HCl (pH 7.5), 5 mM DTT, 10 mM MgCl_2_, 5 μg of full-length Prp28 and increasing amounts of trypsin (0, 1, 2, 10, 50 ng) were incubated for 15 min at room temperature. The reactions were quenched with SDS to final concentration of 1% and polypeptides were resolved by electrophoresis through a 16% polyacrylamide gel containing 0.1% SDS. The gel was soaked in 100 ml of electroblotting buffer (10 mM CAPS, pH 11, 10% methanol) for 5 min at room temperature. Polypeptides were transferred to Sequi-Blot PVDF membrane (Bio-Rad) in electroblotting buffer by electrophoresis at 120 V for 2 h at 4°C. After the transfer, the membrane was rinsed with deionized water and 100% methanol. The membrane was stained with 0.1% Coomassie blue in 40% methanol for 2 min, destained in 50% methanol and rinsed with deionized water. The membrane was air-dried and slices containing full-length Prp28 and the 50-kDa tryptic fragment were excised and analyzed by N-terminal Edman sequencing in the Memorial Sloan-Kettering Microchemistry and Proteomics Core Facility.

### Crystallization of SeMet-Prp28-(127–588)

A protein solution containing 0.23 mM SeMet-Prp28-(127–588), 2 mM AMPPNP, 1.2 mM MgCl_2_ and 0.28 mM 10-mer RNA oligonucleotide 5′-UGGUAUGUUC (containing a consensus yeast 5′ splice site; underlined) was pre-incubated at 4°C for 20 min. Aliquots (2 μl) were mixed with an equal volume of precipitant solution containing 30% (v/v) PEG-400, 0.1 M Hepes-NaOH (pH 7.2–7.8), 0.2 M MgCl_2_, 0.1 M glycine. Crystals grown at 4°C by sitting drop vapor diffusion against the precipitant solution were harvested, cryoprotected by direct transfer to 35% PEG-400, 5% glycerol, 0.1 M Hepes-NaOH (pH 7.2–7.8) 0.2 M MgCl_2_, 0.1 M glycine and then flash frozen in liquid nitrogen.

### Diffraction data collection and structure determination

X-ray diffraction data were collected at the Se anomalous peak wavelength at the Argonne National Laboratory beamline ID-24-C equipped with Pilatus 6M-F detector. Due to the radiation sensitivity of the crystals, we used three different isomorphous crystals to obtain highly redundant data. The crystal-to-detector distance of 540 mm resulted in a resolution cut-off at 2.54 Å. The crystals belonged to space group P3_2_21 with unit cell dimensions consistent with two protomers per asymmetric unit, assuming a solvent content of 55.9%. Indexing, integrating and scaling of the diffraction data were performed using iMosflm and Scala, respectively. Diffraction statistics are compiled in Supplementary Table S1. Twenty-four selenium atom sites were identified using Phenix Autosol (17), affirming that the asymmetric unit contained two Prp28 protomers. SAD phases and initial electron density maps were generated with Phenix Autosol. Phenix Autobuild placed 78% and 53% of the Prp28-(127–588) A and B protomer polypeptides into density, respectively. The model was iteratively adjusted in COOT (18) and refined in PHENIX (17) without using non-crystallographic symmetry restraints. The maps revealed Fo–Fc density for AMPPNP•Mg^2+^ in the active site of the B protomer (Supplementary Figure S1), consistent with exposure of the protein to AMPPNP prior to crystallization, and to MgCl_2_ prior to and during crystallization. No nucleotide or metal ion was observed bound to the A protomer. There was no electron density corresponding to the RNA ligand. The final refined 2.54 Å SeMet-Prp28-(127–588) model comprised a continuous polypeptide from amino acids 134–588 in protomer A and two segments in protomer B, from amino acids 134–468 and 471–583, punctuated by a two-amino acid gap. There was no visible electron density for the N-terminal seven amino acids in either protomer or for the C-terminal five amino acids in protomer B. The refined model had *R*_work_/*R*_free_ values of 0.166/0.219, excellent geometry and no Ramachandran outliers (Supplementary Table S1).

### Yeast *PRP28* expression plasmids

A 2.8-kbp DNA segment bearing the *PRP28* gene was amplified from *S. cerevisiae* genomic DNA by PCR using primers that introduced restriction sites for inserting the gene into the plasmid pRS316 (*CEN URA3*). The resulting plasmid p316-PRP28 includes the *PRP28* ORF plus 520 bp of upstream and 560 bp of downstream genomic DNA. A second yeast expression plasmid, p413-PRP28 (*CEN HIS3*), containing the *PRP28* ORF plus 520 bp of upstream and 350 bp of downstream genomic DNA, was constructed so as to introduce a 5′ BamHI site and a 3′ XhoI site immediately flanking the ORF. Alanine mutations were introduced into the *PRP28* gene by two-stage overlap extension PCR with mutagenic primers. N-terminal truncation alleles *PRP28-(21–588)*, *PRP28-(51–588)*, *PRP28-(77–588)*, *PRP28-(90–588)*, *PRP28-(111–588)*, *PRP28-(123–588)*, *PRP28-(127–588)* and *PRP28-(134–588)* were generated by PCR amplification with sense-strand primers that introduced a BamHI site and a methionine codon in lieu of codons for Leu20, Glu50, Asn76, Asn89, Ile110, Gly122, Lys126 or Met134. The mutated PCR products were digested with BamHI and XhoI and inserted into p413-PRP28 (*CEN HIS3*) in lieu of the wild-type *PRP28* gene. The inserts of each of the pRS413-based plasmids were sequenced completely to confirm that no unwanted coding changes were acquired during amplification and cloning. For inducible expression of *PRP28*, the ORFs encoding wild-type Prp28 and the D341A, E342A, R476A and R264A-R320A mutants were inserted downstream of the *GAL1* promoter in a pRS423-based plasmid (2μ *HIS3*).

### Yeast strains and tests of *PRP28* function *in vivo*

To develop a plasmid shuffle assay for gauging mutational effects on Prp28 function, we generated a *prp28*Δ::*natMX* strain that relies for viability on maintenance of the wild-type *PRP28* gene on a *CEN URA3* plasmid, p316-PRP28. *prp28*Δ [p316-PRP28] cells were unable to grow on medium containing 0.75 mg/ml 5-fluoroorotic acid (FOA). For plasmid shuffle, *prp28*Δ [p316-PRP28] cells were transfected with *CEN HIS3 PRP28* plasmids. Individual His^+^ transformants were selected and streaked on agar medium containing FOA. The plates were incubated at 20, 30 or 37°C and mutants that failed to form macroscopic colonies at any temperature after 8 days were deemed lethal. Individual FOA-resistant colonies with viable *PRP28* alleles were grown to mid-log phase in YPD broth and adjusted to the same *A*_600_ values. Aliquots (3 μl) of serial 10-fold dilutions were spotted to YPD agar plates, which were then incubated at temperatures ranging from 20 to 37°C.

## RESULTS

### Probing Prp28 structure by limited proteolysis

**Figure 1.**
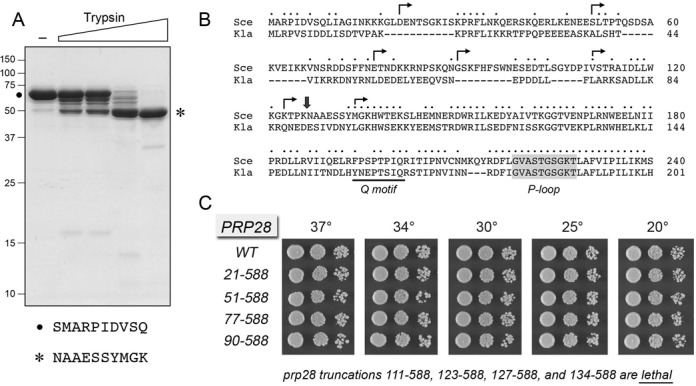
Limited proteolysis of Prp28 and effects of N-terminal truncations on Prp28 activity *in vivo*. (**A**) Limited proteolysis. Reaction mixtures (10 μl) containing 50 mM Tris-HCl (pH 7.5), 5 mM DTT, 10 mM MgCl_2_, 5 μg of full-length Prp28 and increasing amounts of trypsin (0, 1, 2, 10, 50 ng, from left to right) were incubated for 15 min at room temperature. The mixtures were analyzed by SDS-PAGE. The Coomassie blue-stained gel is shown. The positions and sizes (kDa) of marker polypeptides are indicated on the left. The N-terminal sequences of the intact Prp28 polypeptide (•) and the 50-kDa tryptic fragment (asterisk) are shown below the gel. (**B**) The amino acid sequence of the N-terminal segment of *S. cerevisiae* (Sce) Prp28 upstream of Q motif (bracketed) and the P-loop (shaded grey) is aligned to that of the homologous protein from *Kluyveromyces lactis* (Kla). Positions of amino acid site chain identity/similarity are denoted by • above the sequence. Arrowheads indicate the boundaries of the N-terminal deletions of Prp28. (**C**) The wild-type and deleted *PRP28* alleles were tested for activity by plasmid shuffle as described under Materials and Methods. The growth phenotypes of viable FOA-resistant *prp28*Δ p[*CEN HIS3 PRP28*] strains bearing the indicated *PRP28* alleles were assessed as follows. Liquid cultures were grown to mid-log phase and adjusted to the same *A*_600_. Aliquots (3 μl) of serial 10-fold dilutions of cells were spotted to YPD agar. The plates were incubated at the indicated temperatures and photographed after 2 days (30, 34 and 37°C), 3 days (25°C) or 4 days (20°C). The truncated mutants listed at *bottom* failed to complement *prp28*Δ in a plasmid shuffle assay and were deemed lethal.

We produced yeast Prp28 in *E. coli* as an N-terminal His_10_Smt3 fusion and isolated the recombinant protein from a soluble bacterial extract by Ni-affinity chromatography. The His_10_Smt3 tag was removed with the Smt3-specific protease Ulp1, and native Prp28 protein was separated from the tag by a second round of Ni-affinity chromatography. The tag-free Prp28 was further purified by heparin-Sepharose chromatography and gel filtration. Prp28 eluted as single discrete peak during gel filtration at an elution volume consistent with Prp28 being a monomer in solution (not shown). SDS-PAGE verified the purity of the 67 kDa Prp28 polypeptide (Figure 1A, lane –, species denoted by •). Purified Prp28 (5 μg) was subjected to limited proteolysis with 1, 2, 10 and 50 ng of trypsin. Although there are 83 potential tryptic cleavage sites in Prp28 (55 lysines and 28 arginines), only a single major tryptic fragment of 50 kDa was formed under conditions in which all of the input full-length Prp28 was incised (Figure 1A, species denoted by an asterisk). Several polypeptides migrating between 50 kDa and 67 kDa were seen at lower levels of trypsin that did not incise all of the input full-length Prp28. The N-terminal sequences of full-length Prp28 and the 50-kDa tryptic fragment were determined by automated Edman chemistry after transfer of the gel contents to a PVDF membrane. The N-terminus of intact Prp28 (SMARPIDVSQ) initiates with a serine derived from the Ulp1 cleavage site at the tag junction, followed by the native N-terminal methionine of the yeast Prp28 polypeptide. The N-terminal sequence of the 50 kDa fragment (NAAESSYMGK) initiated at Asn127, consistent with tryptic cleavage at Lys126 (Figure 1B, denoted by 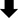). Although we did not directly map the C-terminus of the 50 kDa tryptic fragment, its mobility during SDS-PAGE agrees with that predicted for Prp28-(127–588). Note that we did not detect the accumulation of a stable 14.4 kDa N-terminal tryptic product that would correspond to Prp28-(1–126). We surmise from the limited proteolysis experiment that yeast Prp28 consists of a protease-sensitive N-terminal module preceding a comparatively protease-resistant C-terminal 50 kDa structural domain.

### N-terminal truncations define a minimized functional Prp28 domain

Figure 1B shows an alignment of the N-terminal 240-aa segment of *S. cerevisiae* Prp28 to the homologous N-terminal 201-amino acid segment of Prp28 from the yeast *Kluyveromyces lactis*. The alignment highlights the position of the two most proximal defining motifs of the DEAD-box family: the Q motif that is predicted to contact the adenine nucleobase of ATP and the P-loop motif (also called motif I or the Walker A box) that engages the NTP phosphates (10,11). Whereas the trypsin-sensitive ^126^KN^127^ dipeptide in *S. cerevisiae* Prp28 is not conserved in the *K. lactis* protein, the tryptic site demarcates a transition from a relatively poorly conserved N-terminal segment (36/126 positions of side chain identity/similarity, punctuated by five gaps in the alignment) to a more highly conserved region from Tyr133 to Leu218 immediately preceding the P-loop (62/87 positions of side chain identity/similarity, with no gaps). We used this alignment to guide serial N-terminal truncations of Prp28, at sites denoted by arrows in Figure 1B. The wild-type and truncated alleles were placed on *CEN HIS3* plasmids under the control of the native *PRP28* promoter and tested by plasmid shuffle for complementation of a *prp28*Δ p[*CEN URA3 PRP28*] strain. The resulting *PRP28-(21–588), PRP28-(51–588), PRP28-(77–588)* and *PRP28-(90–588)* strains were viable after FOA selection and grew as well as wild-type *PRP28* cells on YPD agar at all temperatures tested, as gauged by colony size (Figure 1C). The *PRP28-(111–588), PRP28-(123–588), PRP28-(127–588)* and *PRP28-(134–588)* alleles were lethal, i.e. they failed to complement growth of the *prp28*Δ p[*CEN URA3 PRP28*] test strain on FOA at any temperature tested. We conclude that: (i) the N-terminal 89 amino acids are dispensable for Prp28 function *in vivo* and (ii) the segment from aa 90–111 is essential for cell viability.

### Prp28 is an RNA-dependent ATPase

**Figure 2.**
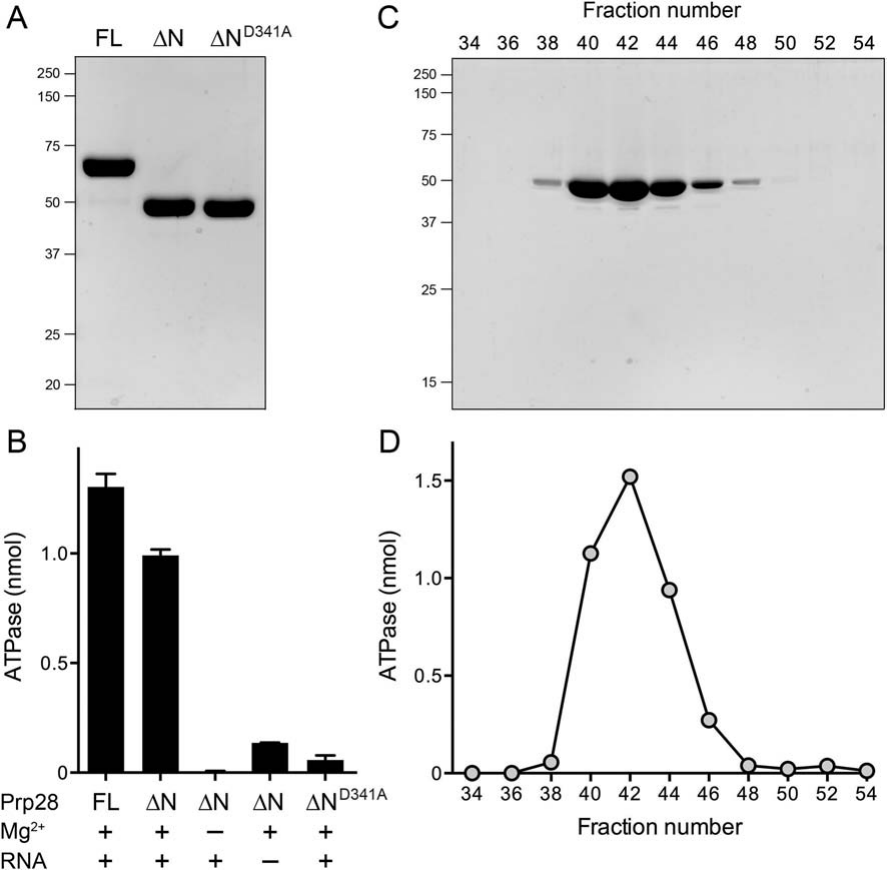
RNA-dependent ATPase activity. (**A**) Recombinant proteins. Aliquots (5 μg) of the Superdex-200 preparations of full-length Prp28 (FL), Prp28-(127–588) (NΔ) and Prp28-(127–588)-D341A (NΔ^D341A^) were analyzed by SDS-PAGE. The Coomassie blue-stained gel is shown. The positions and sizes (kDa) of marker polypeptides are indicated on the left. (**B**) ATPase reaction mixtures (10 μl) containing 20 mM Tris-HCl, pH 7.1, 1 mM (10 nmol) [α^32^P]ATP, 10 mM MgCl_2_ (where indicated by +), poly(U) RNA (200 μM total uridine nucleotide, where indicated by +) and 10 μM (100 pmol) of the indicated Prp28 protein were incubated at 30°C for 60 min. The reactions were quenched by adding 2 μl of 5 M formic acid. An aliquot of the mixture was spotted on a polyethyleneimine-cellulose thin layer chromatography (TLC) plate. Ascending TLC was performed with 0.45 M ammonium sulfate as the mobile phase. [α^32^P]ADP formation was quantified by scanning the TLC plate with a Fujix BAS2500 imager. Each datum in the graph is an average of three separate experiments ± SEM. (**C**) Gel filtration of Prp28-(127–588). Aliquots (1 μl) of the indicated even-numbered Superdex-200 fractions across the elution peak were analyzed by SDS-PAGE. The Coomassie blue-stained gel is shown. The positions and sizes (kDa) of marker polypeptides are indicated on the left. (**D**) Aliquots (1 μl) of the indicated Superdex-200 fractions from panel C were assayed for ATP hydrolysis in reaction mixtures constituted as described in panel B. The ATPase activity profile coincides with the elution profile of the Prp28-(127–588) polypeptide.

Although the trypsin-resistant fragment Prp28-(127–588) did not suffice for activity *in vivo*, we considered it a plausible candidate to comprise a catalytic phosphohydrolase domain, given its N-terminal start site upstream of the Q motif and the P-loop. Therefore, we produced recombinant Prp28-(127–588) in bacteria and purified the tag-free protein (labeled ΔN in Figure 2A) via the protocol developed for full-length Prp28 (labeled FL in Figure 2A). In parallel, we produced and purified a mutated version of Prp28-(127–588) in which the eponymous Asp341 of the ^341^**D**EAD-box motif (also referred to as motif II) was changed to alanine (labeled ΔN^D341A^ in Figure 2A). Reaction of the recombinant FL and ΔN proteins with 1 mM [α^32^P]ATP and 10 mM MgCl_2_ in the presence of poly(U) RNA resulted in the hydrolysis of [α^32^P]ATP to [α^32^P]ADP, with no detectable formation of [α^32^P]AMP (not shown). The extents of ATP hydrolysis by the FL and ΔN Prp28 proteins during a 60 min reaction (1.3 and 1.0 nmol) correspond to the turnover of 13 and 10 ATPs per input enzyme, respectively (Figure 2B). ATP hydrolysis by Prp28-(127–588) was abolished when magnesium was omitted from the reaction mixture and was reduced by 7-fold when poly(U) RNA was omitted (Figure 2B). Mutation of Asp341 to alanine reduced ATP hydrolysis by 17-fold (Figure 2B). The ATPase activity profile coincided with the elution profile of the Prp28-(127–588) polypeptide during gel filtration chromatography (Figure 2C and D). We conclude from these experiments that yeast Prp28 has an intrinsic RNA-dependent ATPase activity, albeit with a low turnover number.

### Crystal structure of yeast Prp28-ΔN

We grew crystals from a mixture of SeMet-substituted Prp28-(127–588), AMPPNP, MgCl_2_ and 10-mer RNA oligonucleotide. The crystals were in space group P3_2_21 with two Prp28 protomers per asymmetric unit. Se-SAD methods were implemented to solve the structure at 2.54 Å resolution (Supplementary Table S1). The maps revealed F_o_–F_c_ density for AMPPNP•Mg^2+^ in the active site of the B protomer (Supplementary Figure S1), but there was no electron density corresponding to the RNA.

**Figure 3.**
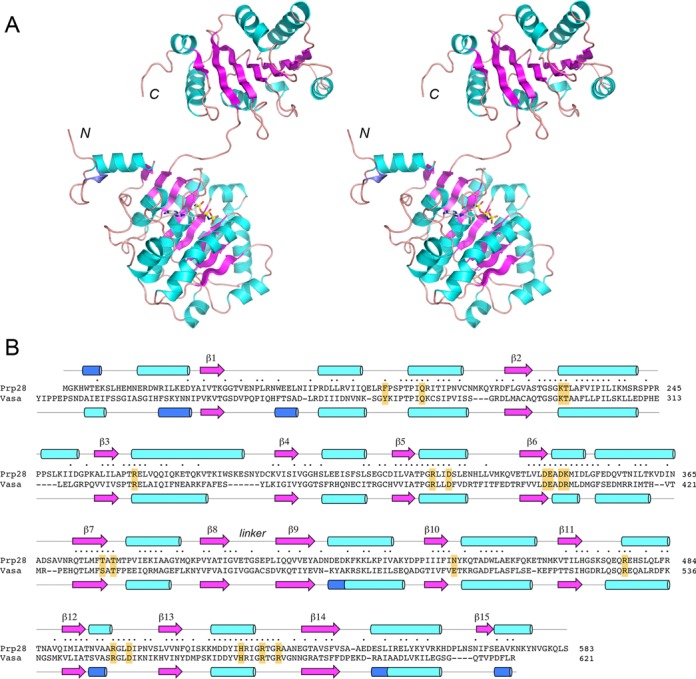
Structure of yeast Prp28-ΔN and homology to Vasa. (**A**) A stereo view of the tertiary structure of the B protomer of Prp28-ΔN is shown as a cartoon model, with β strands colored magenta, α helices colored cyan and 3_10_ helices colored blue. The N and C termini are indicated. AMPPNP bound to the N domain is depicted as a stick model. (**B**) The secondary structure elements of yeast Prp28 and *Drosophila* Vasa (colored as in panel A) are shown above and below their aligned amino acid sequences, with β strands rendered as arrows and helices as cylinders. Gaps in the alignment are indicated by dashes. Positions of amino acid side chain identity/similarity are indicated by • above the sequence. The conserved amino acids in Prp28 that were target for alanine scanning in Prp28 are highlighted in yellow boxes.

The tertiary structure of the B protomer is shown in stereo view in Figure 3A. The secondary structure elements are aligned over the Prp28 amino acid sequence in Figure 3B. Prp28 comprises two structural domains (referred to as RecA-like) that are splayed widely apart. The N domain is organized around an eight-strand β sheet with topology β1↓•β8↑•β2↑•β7↑•β6↑•β3↑•β5↑•β4↑. Five α helices pack against each face of the N domain β sheet. The C domain is built around a seven-strand β sheet with topology β15↑•β9↑•β14↑•β13↑•β10↑•β12↑•β11↑. The C domain β sheet is flanked by five α helices on one side and two α helices on the other side.

A DALI search (19) of the Prp28 N domain structure against the protein database identified many DEAD-box proteins as the top hits, with Z scores from 33.5 to 25.1 (Supplementary Table S2). A separate search with the Prp28 C domain retrieved DEAD-box proteins with Z scores from 23.5 to 17.1 (Supplementary Table S3). As expected, there was substantial overlap in the top hit lists for the two separate domains. The closest homologs of yeast Prp28 include Vasa (16), the canonical DEAD-box translation factor eIF4A (20), RNA folding chaperone Mss116 (21), pre-mRNA splicing factors Prp5 and UAP56 (22,23), exon junction complex subunit eIF4A-III (24,25), mRNA export protein Dbp5 (26), various human DDX proteins (27) and the recently reported human Prp28 homolog (14). An alignment of the secondary and primary structures of yeast Prp28 and *Drosophila* Vasa is shown in Figure 3B. The two N domains superimpose with 2.0 Å rmsd at 228 Cα positions; the C domains superimpose with 2.5 Å rmsd at 161 Cα positions. The Prp28 and Vasa primary structures are related by 202 positions of side chain identity/similarity (Figure 3B, denoted by • above the alignment).

### Flexible domain orientation in Prp28

**Figure 4.**
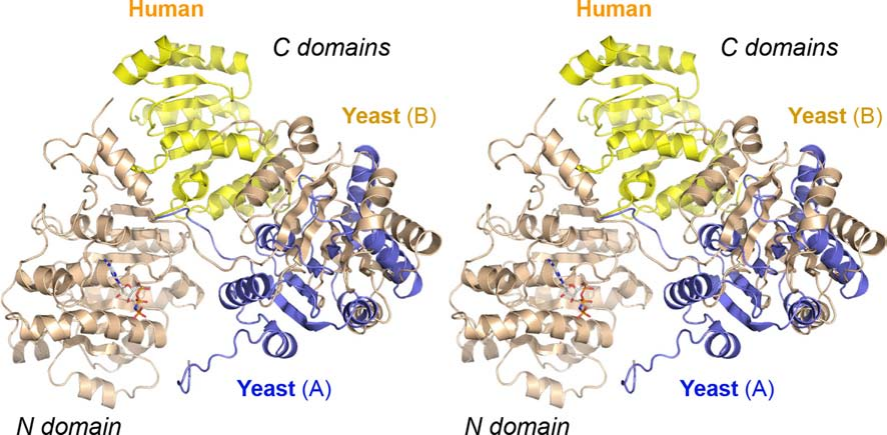
Domain mobility in open conformations of yeast and human Prp28. The stereo image shows the positions of the C domains of the yeast Prp28 A protomer (in blue), yeast Prp28 B protomer (in beige) and human Prp28 (pdb 4NHO, in yellow) when the N domains of the Prp28 polypeptides are superimposed. The N domain is shown in beige for the yeast Prp28 B protomer only. The C domains differ by virtue of large rigid body motions around an interdomain linker segment.

The A and B protomers in the asymmetric unit of yeast Prp28 are virtually identical with respect to the folds of the individual N and C domains. (The N domains superimpose with rmsd of 0.95 Å; the C domains with rmsd of 0.70 Å.) However, the orientation of the N and C domains with respect to each other varies significantly in the two protomers. Figure 4 shows the position of the C domains of the yeast A protomer (in blue) and B protomer (in beige) when the N domains of the two Prp28 polypeptides are superimposed (the N domain is shown in beige for the B protomer only). The C domains differ by virtue of a large rigid body motion around an interdomain linker segment, ^402^GVETSEP^409^ (Figure 3B) that results in a 45 Å displacement of the Cα atom of the Asn577 residue (located at the end of the C-terminal α helix). When we superimpose the N domain of human Prp28 on the yeast Prp28 N domain, we see that the human C domain (colored yellow in Figure 4) adopts yet another orientation, again by virtue of a rigid body movement about the hinge. The position of the human C domain overlaps not at all with the C domains of the yeast A and B protomers. The Pro799 residue in the human Prp28 C domain (the equivalent of yeast residue Asn577) is located 49 Å away from Asn577 in the yeast A protomer and 29 Å away from Asn577 in the yeast B protomer. We conclude that Prp28 can sample a broad range of domain orientations in the absence of a bound RNA and/or interactions with the spliceosome.

### Binding of AMPPNP to Prp28 in a noncatalytic conformation

**Figure 5.**
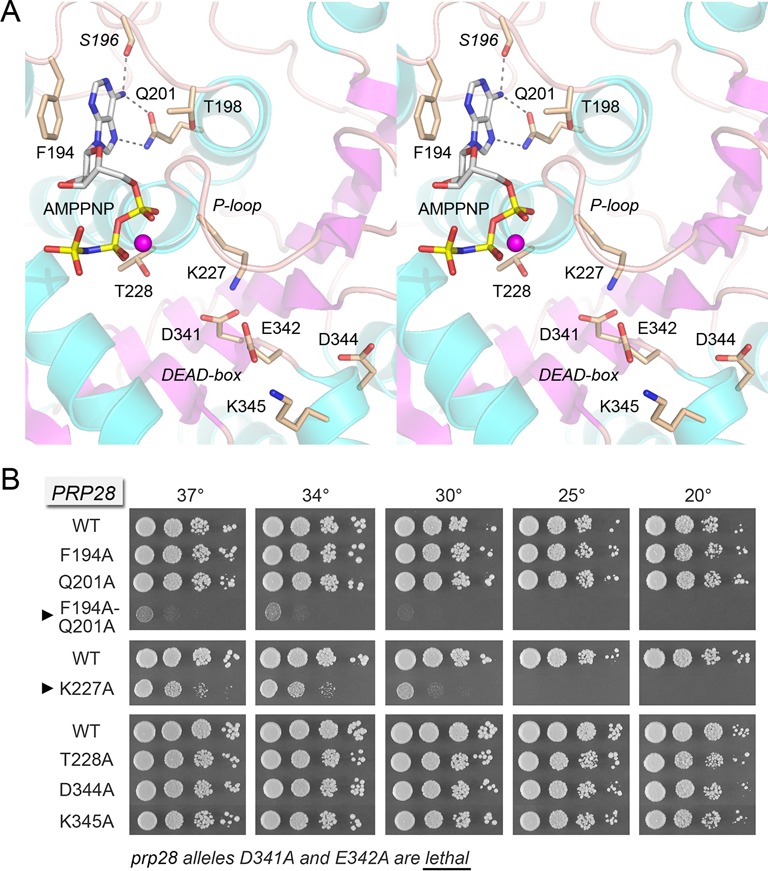
Prp28 binding to AMPPNP and mutational analysis of the binding site_._ (**A**) Stereo view of the ATP site in the N domain of the yeast Prp28-ΔN B protomer. Amino acids and AMPPNP are shown as stick models with beige and gray carbons, respectively. Amino acid side chains are labeled by single letter code and numbered; the *S196* main chain is in italics. Mg^2+^ is depicted as a magenta sphere. The P-loop and DEAD-box motifs are indicated. Hydrogen bonds to the adenine N6 and N7 atoms are denoted by dashed lines. (**B**) The indicated *PRP28* mutant alleles were tested for *prp28*Δ complementation by plasmid shuffle. *D341A* and *E342A* were lethal. The viable FOA-resistant strains were spot-tested for growth on YPD agar at the temperatures specified, in parallel with the isogenic wild-type *PRP28* strain. Alleles with strong cold-sensitive growth defects are indicated by ▸ at *left*.

**Figure 6.**
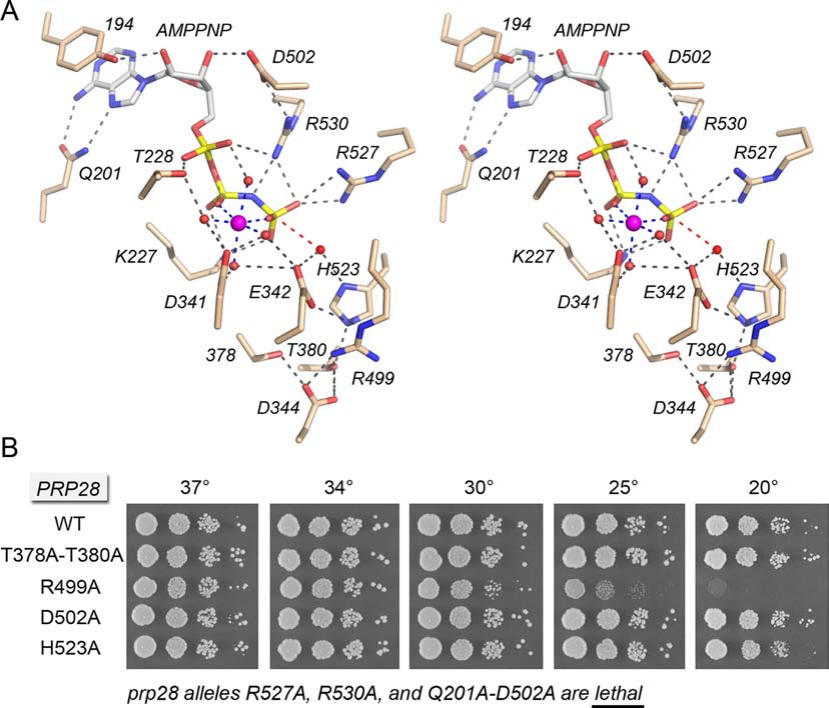
Structure-function analysis of the Prp28 ATPase site guided by the closed-domain Vasa•AMPNP•Mg^2+^ structure. (**A**) Stereo view of the ATP site in the closed domain conformation of Vasa, exemplifying a Michaelis complex with AMPPNP•Mg^2+^. Amino acids and AMPPNP are shown as stick models with beige and gray carbons, respectively. Mg^2+^ is depicted as a magenta sphere in the center of an octahedral coordination complex that bridges the β and γ phosphates. Waters in the metal coordination complex are denoted by red spheres. Atomic contacts are indicated by dashed lines. The in-line orientation of the water nucleophile (red sphere) with respect to the ADP leaving group is denoted by a red dashed line. The conserved amino acids are re-numbered according to their positions in yeast Prp28. (**B**) The indicated *PRP28* mutant alleles were tested for *prp28*Δ complementation by plasmid shuffle. *R527A*, *R530A* and *Q201A-D502A* were lethal. The viable FOA-resistant strains were spot-tested for growth on YPD agar at the temperatures specified, in parallel with the isogenic wild-type *PRP28* strain.

The B protomer of yeast Prp28 was captured in a complex with AMPPNP and Mg^2+^. The nucleotide was bound wholly within the N domain (Figure 3A). A stereo view of the nucleotide binding site is shown in Figure 5A. The adenine nucleobase, in *anti* conformation with respect to the ribose, is sandwiched between Phe194, on which it makes a π stack, and Thr198 Cγ, which makes van der Waals contact to the adenine N6, C5, C6 and N7 atoms. The eponymous Gln201 of the Q motif makes hydrogen bonds with the adenine N6 and N7 atoms that we presume confer adenine specificity. There is an additional hydrogen bond to adenine N6 from the Ser196 main-chain carbonyl (Figure 5A). By reference to other DEAD-box protein structures with bound nucleotide, including Vasa (16; Figure 6A), we infer that the adenine interactions in the Prp28•AMPPNP complex faithfully reflect how the enzyme engages the nucleobase during ATP hydrolysis. By contrast, the position and orientation of the triphosphate moiety of AMPPNP (Figure 5A) are clearly not reflective of a catalytically productive enzyme-substrate complex, insofar as: (i) the nucleotide makes no contact with the P-loop Lys227 side chain, which typically bridges the β and γ phosphates (Figure 6A), but is instead oriented in the opposite direction; (ii) Mg^2+^ bridges the α and β phosphates (Figure 5A), rather than the β and γ phosphates as seen in the Vasa•AMPPNP•Mg^2+^ structure (Figure 6A) and (iii) the divalent cation is remote from the P-loop Thr228 and DEAD-box Asp341 and Glu342 side chains (Figure 5A) that comprise the outer shell of the octahedral metal coordination complex in the Vasa•AMPPNP•Mg^2+^ structure (Figure 6A).

### Effects of mutating the Q motif, P-loop and DEAD-box on Prp28 activity *in vivo*

Q motif (^194^FPSPTPIQ^201^) residues Phe194 and Gln201, P-loop (^221^ASTGSGKT^228^) residues Lys227 and Thr228, and ^341^DEADK^345^ residues Asp341, Glu342, Asp344 and Lys345 were mutated singly to alanine in the context of full-length Prp28. The *PRP28-Ala* alleles were placed on *CEN HIS3* plasmids under the control of the native *PRP28* promoter and tested by plasmid shuffle for *prp28*Δ complementation. The DEAD-box mutations *D341A* and *E342A* were lethal *in vivo* (Figure 5B). The catalytic roles of Asp341 and Glu342 in ATP hydrolysis by Prp28 can be surmised with confidence from the structure of RNA-bound *Drosophila* Vasa in a Michaelis complex with AMPPNP•Mg^2+^, which is depicted in stereo in Figure 6A, with signature amino acids in the ATPase site re-numbered according to their position in yeast Prp28. The equivalent of Asp341, which is critical for Prp28 ATPase *in vitro* (Figure 2B), coordinates three of the waters in the octahedral Mg^2+^ complex with ATP. The equivalent of Glu342 coordinates Mg^2+^-associated waters and the water nucleophile in the phosphohydrolase reaction. Glu342 is presumed to activate the nucleophilic water by serving as a general base catalyst. In the Vasa structure, the water nucleophile is located 3.2 Å from the γ phosphorus (red line in Figure 6A) in an apical orientation with respect to the bridging β­­–γ imido nitrogen atom (the leaving group).

**Figure 7.**
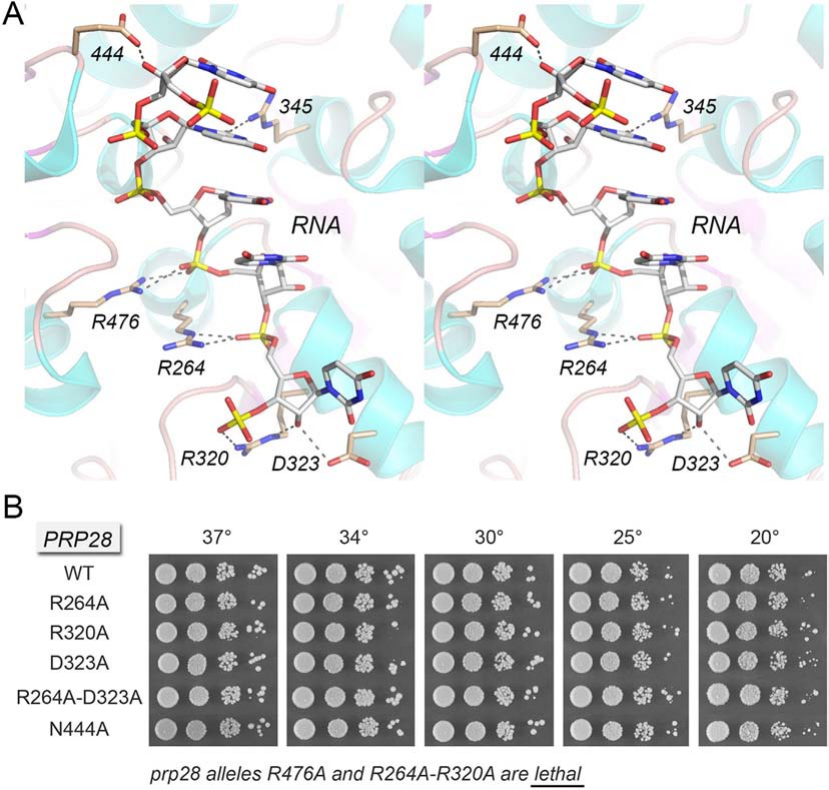
Structure-function analysis of the putative Prp28 RNA site guided by the Vasa•RNA structure. (**A**) Stereo view of the RNA site of Vasa. Amino acids and RNA are shown as stick models with beige and gray carbons, respectively. Atomic contacts are indicated by dashed lines. The conserved amino acids are re-numbered according to their positions in yeast Prp28. (**B**) The indicated *PRP28* mutant alleles were tested for *prp28*Δ complementation by plasmid shuffle. *R476A* and *R264A-R320A* were lethal. The viable FOA-resistant strains were spot-tested for growth on YPD agar at the temperatures specified, in parallel with the isogenic wild-type *PRP28* strain.

By contrast, vicinal DEAD-box mutations *D344A* and *K345A* had no evident impact on cell growth at 20–37°C, as gauged by colony size and number when serial dilutions of these strains were spotted on YPD agar (Figure 5B). The Vasa equivalent of Prp28 Asp344 participates in a hydrogen-bonding network with the two hydroxyamino acid side chains of motif III (^378^TAT^380^ in Prp28; SAT in Vasa) and an arginine from motif Va (^499^RGLDI^503^ in Prp28; same sequence in Vasa). Apparently, these interactions of the distal DEAD-box aspartate are not essential for Prp28 activity *in vivo*. In the yeast Prp28 structure, Lys345 immediately flanking the ^341^DEAD^344^ box makes a salt bridge to Glu342 (Figure 5A). In the Vasa structure, the equivalent residue is an arginine (Arg403; Figure 3B), which makes a hydrogen bond to a uracil nucleobase of the RNA cofactor (Figure 7A). Evidently, the atomic interactions of Prp28 Lys345 are not important for *in vivo* activity.

In the same vein, the Q motif mutations *F194A* and *Q201A* had no effect on yeast growth at 20–37°C (Figure 5B). The tolerance of Prp28 for subtraction of individual adenine-binding Q motif residues Phe194 and Gln201 contrasts with the lethality of the equivalent Gln-to-Ala mutations in the Q motif of the yeast DEAD-box proteins eIF4A and Ded1 and with the severe slow growth and lethal phenotypes, respectively, of the equivalent Phe-to-Ala changes in the Q motif of eIF4A and Ded1 (28,29). We considered the prospect that the adenine contacts of Phe194 and Gln201 might be functionally redundant. To address this point, we tested the *in vivo* activity of a *F194A-Q201A* double mutant. The *F194A-Q201A* strain was barely viable at 34–37°C and failed to thrive at 20 and 25°C (Figure 5B). We surmise that either adenine-binding side chain suffices for a threshold level of Prp28 activity *in vivo*, but loss of both adenine contacts has dire consequences. These results suggest that ATP binding is a critical component of Prp28 function.

To our surprise, replacing the signature P-loop lysine or threonine residues with alanine, which typically abolishes the *in vivo* functions of DEAD/H-box proteins (28,30–33), was not sufficient to abolish the function of Prp28. To wit, the *T228A* change had no effect on cell growth at 20–37°C (Figure 5B). The *K227A* strain was viable though slow-growing at 34–37°C, but failed to form colonies at 20–25°C (Figure 5B).

### Vasa-guided mutagenesis of Prp28 ATPase motifs III, Va and VI

The Vasa structure reveals the disposition of amino acids in the ATPase motifs in a state immediately prior to the chemical step of ATP hydrolysis. Motif III contributes atomic contacts to the DEAD-box, motif Va and the histidine side chain of motif VI (^523^HRIGRTGR^530^ in Prp28; same sequence in Vasa) (Figure 6A). The motif III hydroxyamino acids of other exemplary DExD/H proteins are dispensable for NTP hydrolysis, but critical for the coupling of hydrolysis to duplex unwinding (31,33,34). Motif III mutations elicit variable *in vivo* phenotypes in different DExD/H proteins, ranging from lethality to conditional (*ts/cs*) growth defects to no effect on cell growth (30,31,33,35,36). Here we found that the Prp28 motif III double-alanine mutation *T378A-T380A* had no effect on cell growth at 20–37°C (Figure 6B). This result hints that Prp28 might not be acting as a vigorous translocase/helicase in executing its essential function in pre-mRNA splicing *in vivo*.

Motif Va contributes two amino acids to the ATPase active site in Vasa. The equivalent of Prp28 Asp502 makes a hydrogen bond with the ribose 3′-OH of ATP and forms a salt bridge to the motif VI arginine corresponding to Prp28 Arg530 (Figure 6A). We found that the Prp28 *D502A* mutation had no effect on yeast growth at 20–37°C (Figure 6B). We considered the prospect that the Asp502 interaction with the adenosine nucleoside might be functionally redundant with a contact to the adenine nucleobase, a suspicion that was verified by the synthetic lethality of a double mutation in which Asp502 and the Q motif Gln201 were simultaneously changed to alanine (Figure 6B). The Vasa equivalent of Prp28 motif Va residue Arg499 makes salt bridges to DEAD-box side chains Glu342 and Asp344 (Figure 6A). Here we observed that replacing Prp28 Arg499 with alanine had no effect on yeast growth at 30–37°C, but elicited a progressively severe cold-sensitive growth defect at 25 and 20°C (Figure 6B).

Motif VI is the principal contribution of the distal RecA-like domain of DExD/H NTPases to the phosphohydrolase active site, formation of which entails closure of the N and C domains around the triphosphate group, as observed in the Vasa structure (Figure 6A). In the yeast Prp28 structure, motif VI and the C domain are located far away from the AMPPNP bound to the N domain (Figure 3A). We presume that the open domain arrangement accounts for the noncatalytic conformation of the AMPPNP triphosphate and metal moieties in the Prp28 structure. In the NTP-bound structures of Vasa (16) and other DExD/H proteins (21,24–26,37), the equivalents of Prp28 motif VI Arg527 and Arg530 coordinate the γ phosphate oxygens (Figure 6A) and, it is presumed, stabilize the transition state of the phosphohydrolase reaction. Replacing yeast Prp28 motif VI residues Arg527 and Arg530 with alanine was unconditionally lethal *in vivo* (Figure 6B). The Vasa motif VI histidine (equivalent to His523 in Prp28) coordinates the nucleophilic water and makes a hydrogen bond to the motif III threonine (Thr380 in Prp28) (Figure 6A). Yet, we found that mutating His523 to alanine in yeast Prp28 had no effect on cell growth at 20–37°C (Figure 6B). Collectively, the results of the mutational analysis of the Prp28 ATPase site suggest that the binding of ATP•Mg^2+^ in a catalytically competent closed conformation is necessary for Prp28 function *in vivo*. The results are consistent with a requirement for ATP hydrolysis, albeit perhaps not at a vigorous level, insofar as *in vivo* function is not compromised by subtraction of contacts made by Thr228 to ATP•Mg^2+^ and by His523 to the water nucleophile.

### Vasa-guided mutagenesis of Prp28 RNA-binding motifs Ia, Ic, IV and IVa

The Vasa structure (16) revealed the basis for single-strand RNA binding by DEAD-box proteins (21,24–26). Constituents of the RNA-binding site are derived from motif Ia (^262^PTREL^266^ in Prp28; same sequence in Vasa), and motif Ic (^317^TPGRLID^323^ in Prp28; TPGRLLD in Vasa) in the N domain and from motif IV (^440^IIFINYK^446^ in Prp28; IVFVETK in Vasa) and motif IVa (^468^HGSKSQEQR^476^ in Prp28; HGDRLQSQR in Vasa) in the C domain. Figure 7A shows a stereo view of the RNA-bind site of Vasa; the conserved amino acids that contact RNA are re-numbered according to their position in yeast Prp28. As discussed in detail previously (16), most of the RNA interactions are with the sugar-phosphate backbone. Here we tested the effects of alanine substitutions for Prp28 RNA-binding residues Arg264 (motif Ia), Arg320 and Asp323 (motif Ic), Asn444 (motif IV) and Arg476 (motif IVa). The salient findings were that the *R264A*, *R320A*, *D323A* and *N444A* mutations had no effect on yeast growth at 20–37°C (Figure 7B). By contrast, the *R476A* mutation was uniquely lethal among the single-alanine changes tested. The equivalents of Prp28 Arg476, Arg264 and Arg320 make electrostatic contacts to three sequential RNA phosphodiesters in the Vasa structure (Figure 7A). Again, we considered the possibility that the backbone interactions of Arg264 and Arg320 might be functionally redundant and, indeed, this appeared to be the case, insofar as the Prp28 *R264A-R320A* double mutant was lethal (Figure 7B). These results indicate that RNA binding is critical for Prp28 function *in vivo*. The Vasa equivalent of the nonessential Prp28 motif Ic residues Arg320 and Asp323 both form hydrogen bonds to the ribose 2′-OH at the 3′ end of the RNA ligand (Figure 7A). To gauge whether loss of one of the ribose 2′-OH contacts synergized with loss of Arg264, we tested a Prp28 *R264A-D323A* double mutation, but observed no effect on yeast growth (Figure 7B).

### Allele-specific dominant-negative effects of overexpressing Prp28 mutants

**Figure 8.**
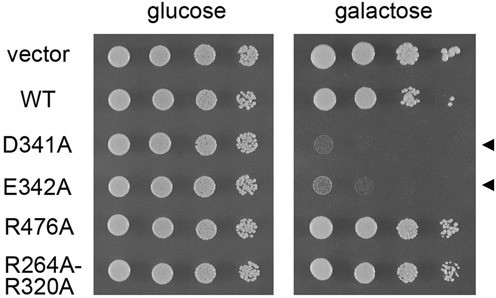
Dominant-negative Prp28 mutations. *PRP28* cells were transformed with 2μ *HIS3* vector or 2μ *HIS3* plasmids bearing the indicated *PRP28* alleles under the transcriptional control of the *GAL1* promoter. Liquid cultures derived from single transformants were grown to mid-log phase at 30°C in SD-His glucose medium and adjusted to the same *A*_600_. Aliquots (3 μl) of serial 10-fold dilutions of cells in water were spotted to SD-His agar with 2% glucose or 2% galactose as indicated. The plates were photographed after incubation at 30°C for 2 days (glucose) or 4 days (galactose).

Schwer *et al.* (30–32) have shown that overexpression of certain non-viable alleles of the DExH-box NTPase splicing factors *PRP16*, *PRP22* and *PRP43* exert severe dominant-negative growth defects in wild-type *PRP16*, *PRP22* and *PRP43* cells. The basis of these effects is that mutations that impair ATP hydrolysis, but do not affect interaction of the NTPase with the spliceosome complex on which they act, will allow the mutant protein to compete with the wild-type NTPase for spliceosome access and thereby freeze the spliceosome in an inactive state (32,38). Here we took a similar approach to query whether non-functional alleles of *PRP28* elicit a dominant-negative phenotype. Wild-type *PRP28* cells were transformed with 2μ *HIS3* plasmids bearing wild-type *PRP28* or the *D341A*, *E342A*, *R476A* and *R264A-R320A* mutants under the transcriptional control of the glucose-repressed, galactose-inducible *GAL1* promoter. Serial dilutions of the His^+^ transformant strains were spotted on SD-His agar plates containing either glucose or galactose. All strains grew equally well on glucose medium when the *GAL1* promoter was off (Figure 8). The instructive findings were that induced overexpression of the *D341A* and *E342A* alleles with loss-of-function mutations in the ATPase active site caused a severe dominant-negative growth defect on galactose medium (Figure 8). The defective motif VI alleles *R527A* and *R530A* and the defective *Q201A-D502A* double mutant also elicited dominant-negative phenotypes when overexpressed (not shown). By contrast, the loss-of-function mutations *R476A* and *R264A-R320A* that perturbed the RNA-binding site had no dominant-negative effect on growth (Figure 8). This is not a trivial consequence of a failure to overproduce the R476A and R264A-R320A proteins. To wit, we gauged the steady-state levels of wild-type and mutant Prp28 proteins by western blot analysis of total cell protein from yeast 2μ *HIS3 PRP28* cells harvested 4 h after transfer from glucose to galactose medium (Supplementary Figure S2). Immunoblotting with anti-Prp43 antibody served as a loading control. Probing with anti-Prp28 antibody verified that cells bearing the wild-type 2μ *HIS3 PRP28* plasmid were induced to accumulate very high levels of Prp28 after transfer to galactose, compared to the scant level of Prp28 seen in cells carrying the empty 2μ *HIS3* vector (Supplementary Figure S2). The instructive findings were that every one of the ‘lethal’ Prp28 mutants also accumulated to similar high levels after galactose induction (Supplementary Figure S2). The results of the dominant-negative experiments suggest that: (i) RNA binding is necessary for Prp28 access to the splicing machinery and (ii) ATP hydrolysis is necessary for Prp28 to disengage from the spliceosome.

## DISCUSSION

The present study comprises a structural, genetic and biochemical analysis of the essential yeast pre-mRNA splicing factor Prp28. We provide what is, to our knowledge, the first evidence that purified Prp28 has an intrinsic RNA-dependent ATP phosphohydrolase activity, albeit a rather feeble one with respect to ATP turnover. A low-powered ATPase activity might well suffice for Prp28 to perform its function in exchanging U1 snRNP and U6 snRNP at the 5′ splice site of yeast pre-mRNAs, e.g. if Prp28 acts as an ATP-driven switch rather than as a translocating motor. It is now established that ATP binding, without hydrolysis, can suffice for some DEAD-box proteins to elicit local RNA or RNP distortion, while hydrolysis serves to re-cycle the protein (10,11,21). Alternatively, the ATPase activity of yeast Prp28 might be invigorated in the context of the spliceosome, e.g. via the influence of specific RNA elements or protein–protein interactions.

The crystal structure of yeast Prp28 verifies that the protein binds ATP within its N-terminal RecA-like domain, with proper and specific contacts to the adenine nucleobase. The wide-open domain conformation we see in the absence of a bound RNA suggests that RNA engagement is needed in order to trigger domain closure and capture of the triphosphate•Mg^2+^ complex in a productive orientation by motif VI of the C domain. The individual N and C domains are structurally quite similar to those of other DEAD-box proteins for which catalytically productive closed complex structures with ATP analogs and RNA have been solved. An appealing scenario for Prp28 is that domain closure is stimulated or stabilized by components of the yeast spliceosome, akin to the mRNA-locked state of ATP-bound eIF4A-III in the context of the exon junction complex (24,25), and that ATP hydrolysis is triggered at the time of U1 snRNP ejection when Prp28 releases its grip on the spliceosome.

Deletion analysis and alanine scanning revealed a high tolerance for subtraction of Prp28 structural elements, whereby removal of the N-terminal 89 amino acids had no impact on yeast growth, nor did alanine substitutions for several of the signature constituents of the phosphohydrolase active site motifs or RNA-binding motifs of the DEAD-box protein family. We surmise that some of the atomic contacts in the ATP- or RNA-binding sites are functionally redundant *in vivo* via observations of synthetic lethality of double-alanine mutations. As mentioned above, Prp28 displays distinctive structure-activity relations *vis à vis* other well-studied DEx(D/H) NTPases, particularly with respect to the inessentiality of the P-loop threonine and the viability of the P-loop lysine mutant. Of the 20 amino acids in Prp28 that were subjected to alanine scanning, only five proved to be unconditionally essential for function *in vivo*: Asp341, Glu342, Arg476, Arg527 and Arg530. In an early mutational study of yeast Prp28, focused primary on screening for conditional (*ts*/*cs*) alleles with libraries of *PRP28* genes randomly mutagenized by error-prone PCR or hydroxylamine, Chang *et al.* (39) identified many growth-defective lesions located within or near the DEAD-family motifs. They also performed site-specific mutagenesis of 10 amino acids. However, most of the molecular lesions were uninformative with respect to structure-activity relations, insofar as they entailed structurally drastic amino acid changes (Ala-to-Val, Gly-to-Val, Gly-to-Glu, Arg-to-Glu, Arg-to-Asp, Ala-to-Trp, etc.). Nonetheless, several of their findings add to the alanine scan performed here, as follows. We found that the motif VI *R527A* allele is lethal. Chang *et al.* noted that *R527K* had no effect on growth. Thus, positive charge at this motif VI side chain, which makes a bifurcated contact to one of the ATP γ phosphate oxygens, suffices for Prp28 function. We found that the motif Va *R499A* change conferred a *cs* growth phenotype. Chang *et al.* reported that *R499G* was lethal (consistent with glycine being more disruptive of local structure than alanine) and that *R499K* was *cs* (mimicking the alanine change). Whereas, we found that motif Va allele *D502A* was benign, Chang *et al.* reported that the conservative *D502N* change was lethal. It is possible that the amide Nδ creates a clash with the nearby essential Arg530 side chain, which in Vasa forms a salt bridge to the equivalent of Asp502. Whereas we see that the motif Ia *R264A* change has no effect on growth, they constructed *R264E* and *R264D* alleles and found that they were both lethal. The upshot of our study is that the RNA phosphate contacts of Arg264 are dispensable (because Arg264 is redundant with Arg320); the findings of Chang *et al.* highlight that charge repulsion between motif Ia and the RNA backbone is deleterious.

In conclusion, the present work establishes a biochemical, structural and genetic framework for dissecting the action of yeast Prp28 during pre-mRNA splicing. ATPase-defective, dominant-negative variants of Prp28 should prove useful in freezing spliceosomes *in vivo* and *in vitro* at the stage where ATP hydrolysis occurs. A key enabling step in understanding Prp28 function will be the development of an *in vitro* splicing system that is completely dependent on exogenous recombinant Prp28 protein.

## ACCESSION NUMBER

The coordinates for the Prp28 structure have been deposited in the RCSB protein structure database (pdb ID code 4W7S).

## SUPPLEMENTARY DATA

Supplementary Data are available at NAR Online.

SUPPLEMENTARY DATA
