# Supplementary material for: Crystal structure, mutational analysis and RNA-dependent ATPase activity of the yeast DEAD-box pre-mRNA splicing factor Prp28
Source: Nucleic Acids Res. 2014 Oct 10;42(20):12885–98. doi: 10.1093/nar/gku930 (PMC4227776; doi:10.1093/nar/gku930)
Supplement: SUPPLEMENTARY DATA [file supp_42_20_12885__index.html]

Crystal structure, mutational analysis and RNA-dependent ATPase activity of the yeast DEAD-box pre-mRNA splicing factor Prp28 — Crystal structure, mutational analysis and RNA-dependent ATPase activity of the yeast DEAD-box pre-mRNA splicing factor Prp28 — SUPPLEMENTARY DATA 

# Crystal structure, mutational analysis and RNA-dependent ATPase activity of the yeast DEAD-box pre-mRNA splicing factor Prp28

## SUPPLEMENTARY DATA

**Files in this Data Supplement:**

- SUPPLEMENTARY DATA
