# Supplementary material for: Crystal structure, mutational analysis and RNA-dependent ATPase activity of the yeast DEAD-box pre-mRNA splicing factor Prp28
Source: Nucleic Acids Res. 2014 Oct 10;42(20):12885–98. doi: 10.1093/nar/gku930 (PMC4227776; doi:10.1093/nar/gku930)
Supplement: SUPPLEMENTARY DATA [file supp_gku930_nar-02362-m-2014-File010.pdf]

Supplemental Material

Crystal structure, mutational analysis, and RNA-dependent ATPase activity of the yeast DEAD-box pre-mRNA splicing factor Prp28

Agata Jacewicz, Beate Schwer, Paul Smith, and Stewart Shuman

Tables S1, S2 and S3

Figures S1 and S2

Table S1

Prp28 crystallographic data and refinement statistics

**Diffraction data**	
Space group	*P*3_2*21*
Cell dimensions	
a, *b*, *c* (Å)	114.72, 114.72, 156.19
α, β, γ (°)	90.00, 90.00, 120.00
Number of crystals	3
Wavelength (Å)	0.9792
Resolution (Å)	57.00 − 2.54 (2.68 − 2.54)
R_{sym} [a]	0.066 (0.253) [60.00 – 2.55]
	0.064 (0.293) [65.00 – 2.54]
	0.058 (0.239) [63.00 – 2.54]
R_{cryst} [a]	0.038 (0.159)
Unique reflections [b]	39724
I / σI	16.7 (5.0)
Completeness (%)	99.8 (99.4)
Multiplicity	20.5 (19.4)
**Phasing**	
Phasing method	Se-SAD (24 Se sites)
Resolution (Å)	53.85-3.30
Figure of merit [c]	0.476
Signal to noise [d]	Ano=0.135
**Refinement statistics** (F > 0)	
Resolution (Å)	53.84 – 2.54 (2.61 – 2.54)
Completeness	99.9 (99.0)
R_{work} / R_{free} [e]	16.6 / 21.9 (19.9 / 27.5)
B-factors ($Å^2$) Overall / Wilson	33.6 / 33.0
RMS deviations	
Bond lengths (Å)	0.009
Bond angles (°)	1.214
Ramachandran plot	
Favored (%)	98.1
Outliers (%)	0
**Model contents**	
Protomers / ASU [f]	2
Protein residues	903
Waters	249
Ligands/Ions	1 AMP-PNP, 2 Mg^{2+}, 8 PEG, 1 glycerol
**PDB ID**	4W7S

a) R_{sym} and R_{cryst} are defined as Rpim and computed in SCALA from a single crystal and from all merged data, respectively. The figures within brackets reflect the resolution ranges for each individual crystal.
b) Friedel's pairs were treated separately
c) Signal to noise for anomalous phasing (Ano) is the fraction of the data for which D_{ano} > 3.0σ D_{ano} condition holds as calculated in Phenix.Xtriage.
d) Figure of merit (FOM) value is an output from Phaser (in Phenix.Autosol)
e) R_{free} set consists of 4.9% of data against which the structure was not refined
f) NCS restrains were not used during refinement.

<u>Table S2</u>

Structural Homologs of Yeast Prp28 N-domain (aa 134-402)

Protein	pdb ID	Z score	Root mean square deviation
Human Prp28	4NHO	33.5	1.8 Å at 254 Cα positions
Human DDX5	3FE2	31.6	1.8 Å at 233 Cα positions
Yeast Prp5	4LK2	30.6	2.0 Å at 240 Cα positions
Human DDX3X	2I4I	29.6	1.9 Å at 225 Cα positions
Drosophila Vasa	2DB3	28.3	2.0 Å at 228 Cα positions
Human DDX52	3DKP	27.9	2.2 Å at 229 Cα positions
Yeast eIF4A	1FUU	27.8	1.8 Å at 213 Cα positions
Thermus thermophilus Hera	4KBG	27.8	1.5 Å at 205 Cα positions
Bombyx mori Vasa	4D26	27.5	2.3 Å at 231 Cα positions
Human DDX47	3BER	27.3	1.9 Å at 211 Cα positions
Human eIF4A	2G9N	27.3	1.9 Å at 213 Cα positions
Human DDX53	3IUY	27.2	1.6 Å at 205 Cα positions
Human DDX18	3LY5	27.0	1.7 Å at 208 Cα positions
Human DDX6	4CT4	26.8	1.9 Å at 209 Cα positions
Human eIF4A-III	4C9B	26.7	2.0 Å at 218 Cα positions
Yeast Mss116	3I5Y	25.8	2.4 Å at 225 Cα positions
Human DBP5	3FHC	25.8	2.3 Å at 217 Cα positions
Yeast Dhh1	1S2M	25.7	1.9 Å at 203 Cα positions
Human DDX20	3B7G	25.1	1.8 Å at 204 Cα positions

Table S3

Structural Homologs of Yeast Prp28 C-domain (aa 403-583)

Protein	pdb ID	Z score	Root mean square deviation
Human DDX41	2P6N	23.4	1.5 Å at 158 Cα positions
Human DDX3X	2JGN	21.8	1.8 Å at 158 Cα positions
Drosophila Vasa	2DB3	20.6	2.5 Å at 161 Cα positions
Human Prp28	4NHO	20.5	2.4 Å at 157 Cα positions
Bombyx mori Vasa	4D25	20.4	2.5 Å at 161 Cα positions
Thermus thermophilus Hera	4KBG	20.4	2.6 Å at 156 Cα positions
Yeast Dbp5	3RRN	19.8	2.9 Å at 158 Cα positions
E. coli SrmB	2YJT	19.7	2.2 Å at 157 Cα positions
Human DDX6	4CRW	19.5	2.2 Å at 150 Cα positions
Human DBP5	3FHT	18.7	2.7 Å at 156 Cα positions
Yeast Prp5	4LK2	18.4	2.6 Å at 166 Cα positions
Human eIF4A-III	2HYI	18.0	2.9 Å at 158 Cα positions
Human UAP56	1T5I	17.9	2.4 Å at 150 Cα positions
Human DDX25	2RB4	17.6	2.2 Å at 147 Cα positions
Yeast eIF4A	1FUK	17.3	2.6 Å at 145 Cα positions
Yeast Dhh1	1S2M	17.1	2.3 Å at 146 Cα positions

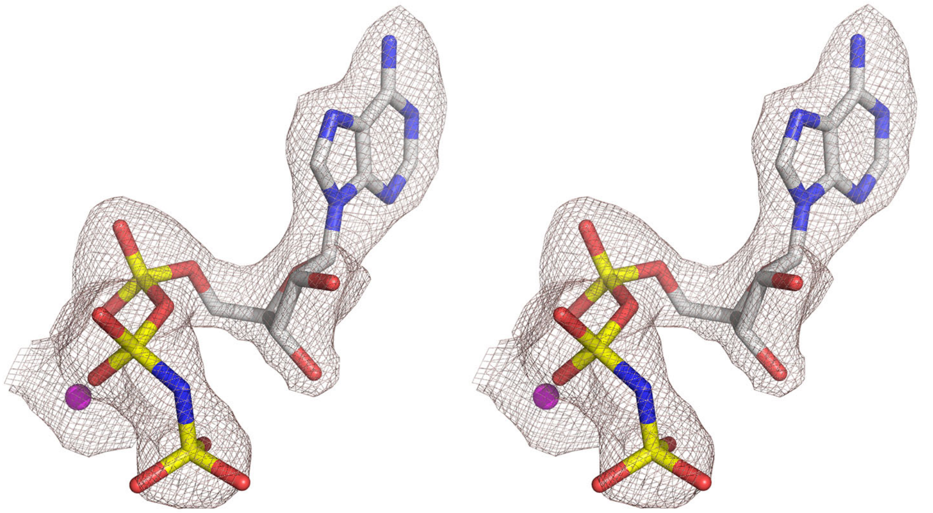

Figure S1. **Electron density for AMPPNP•Mg^{2+}**. Stereo view of the Fo-Fc map (mesh) of the nucleotide in the active site of the B protomer, contoured at 2 σ. The AMPPNP modeled into the density is shown as a stick model. Mg^{2+} is depicted as a magenta sphere.

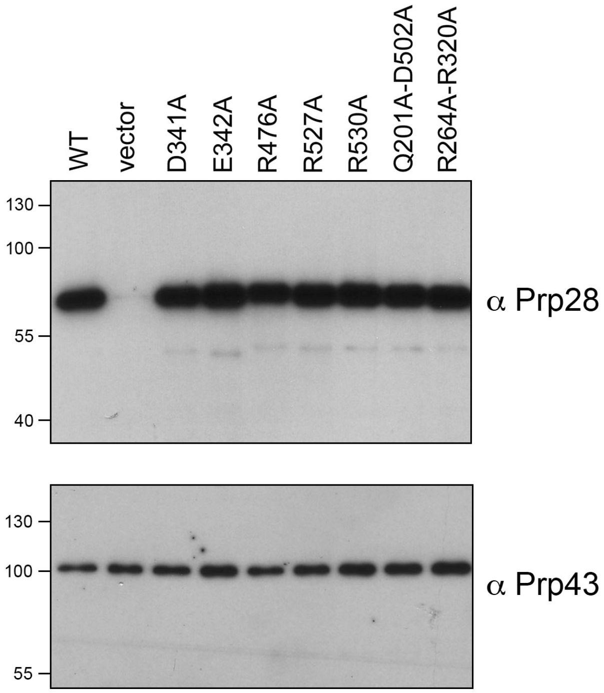

Figure S2. **Immunoblot analysis of steady-state levels of mutant Prp28 proteins**. Wild-type cells bearing 2μ *HIS3* plasmids for expression of the indicated *PRP28* alleles under the control of a *GAL1* promoter, or an empty 2μ *HIS3* vector control, were grown in glucose-containing medium at 30°C until the A_{600} reached ~0.6. Cells were washed with water, suspended in SD-His medium containing 2% galactose at an A_{600} of 0.6 and incubated for 4 h at 30°C. Aliquots (10 A_{600} units) of the cells were collected by centrifugation and lysed in 20% trichloroacetic acid. Total acid-insoluble protein was recovered and resuspended in 1 M Tris-HCl (pH 8.0). Aliquots of the samples, adjusted to the same total protein content based on A_{280} of the extracts, were adjusted to 2% SDS and 0.1 M DTT and then analyzed by electrophoresis through 8% polyacrylamide gels containing 0.1% SDS. The gel contents were transferred to PVDF membranes and the membranes were probed by Western blotting with rabbit polyclonal antibodies against Prp28 or Prp43 as specified. Immune complexes were visualized using horseradish peroxidase-linked anti-rabbit IgG and an ECL Western detection system (Amersham Biosciences, GE Healthcare). The positions and sizes (kDa) of marker polypeptides are indicated on the *left*.
